# Glucose transporter 3 is a rab11-dependent trafficking cargo and its transport to the cell surface is reduced in neurons of CAG140 Huntington’s disease mice

**DOI:** 10.1186/s40478-014-0178-7

**Published:** 2014-12-20

**Authors:** Hollis McClory, Dana Williams, Ellen Sapp, Leah W Gatune, Ping Wang, Marian DiFiglia, Xueyi Li

**Affiliations:** Department of Neurology, Massachusetts General Hospital and Harvard Medical School, Charlestown, MA 02129 USA; Department of Neurology, The Second Hospital of Shandong University, Jinan, Shandong 250033 China

**Keywords:** Huntington’s disease, Glucose transporter 3, Rab11, Recycling endosomes

## Abstract

Huntington’s disease (HD) disturbs glucose metabolism in the brain by poorly understood mechanisms. HD neurons have defective glucose uptake, which is attenuated upon enhancing rab11 activity. Rab11 regulates numerous receptors and transporters trafficking onto cell surfaces; its diminished activity in HD cells affects the recycling of transferrin receptor and neuronal glutamate/cysteine transporter EAAC1. Glucose transporter 3 (Glut3) handles most glucose uptake in neurons. Here we investigated rab11 involvement in Glut3 trafficking. Glut3 was localized to rab11 positive puncta in primary neurons and immortalized striatal cells by immunofluorescence labeling and detected in rab11-enriched endosomes immuno-isolated from mouse brain by Western blot. Expression of dominant active and negative rab11 mutants in clonal striatal cells altered the levels of cell surface Glut3 suggesting a regulation by rab11. About 4% of total Glut3 occurred at the cell surface of primary WT neurons. HD^140Q/140Q^ neurons had significantly less cell surface Glut3 than did WT neurons. Western blot analysis revealed comparable levels of Glut3 in the striatum and cortex of WT and HD^140Q/140Q^ mice. However, brain slices immunolabeled with an antibody recognizing an extracellular epitope to Glut3 showed reduced surface expression of Glut3 in the striatum and cortex of HD^140Q/140Q^ mice compared to that of WT mice. Surface labeling of GABAα1 receptor, which is not dependent on rab11, was not different between WT and HD^140Q/140Q^ mouse brain slices. These data define Glut3 to be a rab11-dependent trafficking cargo and suggest that impaired Glut3 trafficking arising from rab11 dysfunction underlies the glucose hypometabolism observed in HD.

## Introduction

Huntington’s disease (HD) is a progressive neurodegenerative disorder caused by a mutation in huntingtin (Htt) [1]. How mutant Htt leads to neurodegeneration is still under investigation, but may involve defects associated with multiple pathways [2]. Positron emission tomography brain imaging with [^18^ F]-fluorodeoxyglucose has revealed a regional decrease of glucose usage in the striatum (caudate and putamen) and cortex (frontal and temporal lobes) of persons symptomatic and at risk for HD [3-8]. Decreased activity of brain glucose metabolism correlates with the progression of HD [9]. However, the mechanism underlying reduced glucose usage is poorly understood.

Glucose is a polar molecule; its transport across plasma membranes is facilitated largely via the SLC2 family of 13 glucose transporters (Glut). Brain tissues express 8 Glut isoforms, two of which, namely Glut1 and Glut3, are believed to handle the majority of glucose uptake in the brain [10-12]. Glut1 is present in vascular structures across all brain areas including the white matter and localized selectively to astrocytes and microvessel endothelial cells [13,14]. Glut3, on the other hand, is expressed in neurons in all layers of neocortex, the striatum, the thalamus, midbrain, cerebellum and the ependymal layer of all ventricles [14-16]. In the brain and in cultured neurons, Glut3 protein is detected in somata and neural processes [12,15]. HD patients at early stages of striatal degeneration (grade I [17]) have normal levels of Glut3 protein, but show a decline in glucose utilization in the brain [3,8,18]. This suggests that reduced glucose utilization in early stages of HD is unlikely to result from altered expression of Glut3 protein.

Rab11 is a ras-like GTPase that plays a pivotal role in sustaining the homeostatic abundance of receptors, transporters and other critical molecules on the cell surface by regulating vesicle formation at the recycling endosome, vesicle transport along cytoskeleton networks, and vesicle fusion with the plasma membrane [19-22]. The HD mutation is known to reduce the production of active rab11, thereby slowing endosomal recycling of receptors and transporters to the cell surface [23-26]. In a previous study we showed that primary HD^140Q/140Q^ cortical neurons have a deficit in taking up glucose, which can be attenuated upon expression of active rab11 [25]. In this study, we addressed if Glut3 trafficking is regulated by rab11 and if there is a deficit of Glut3 trafficking in HD neurons. We found Glut3 co-distributed with rab11 and present in immuno-isolated brain rab11 enriched endosomes. Glut3 expression on the cell surface of immortalized striatal cells was altered upon expressing mutant forms of rab11. Analysis of the surface expression of Glut3 by biotin labeling and immunostaining with an antibody recognizing an extracellular epitope of Glut3 showed that expression of the transporter on the cell surface was reduced in cultured HD^140Q/140Q^ neurons and in adult HD^140Q/140Q^ mouse brain neurons respectively compared to WT. Our study suggests that defective Glut3 trafficking arising from compromised activation of rab11 in HD neurons contributes to glucose hypometabolism in HD.

## Materials and methods

### Preparation and culture of primary cortical neurons

Homozygous Q140 knock-in HD (HD^140Q/140Q^) and WT mice were maintained and bred at the Animal Core Facility of the Massachusetts General Hospital. Experiments involved in the use of mice were performed according to the institutional and US National Institute of Health guidelines and approved by the Massachusetts General Hospital Subcommittee on Research Animal Care. Neurons from embryonic cortex of mice were isolated and cultured as described previously [25]. In brief, embryos were harvested at embryonic day 16 to 18 (E16 - E18). Cortices were dissected free of meninges and other connective tissues, pooled and incubated in PBS supplemented with penicillin, streptomycin, neomycin, and 0.25% trypsin in a 37°C cell culture incubator for 10 min. Cells released from cortices were washed twice in PBS with calcium and magnesium, and resuspended in NBM media (Neurobasal DMEM containing B27 supplement, N2 supplement, 25 mM mercaptoethanol and L-glutamine), and cultured in poly-L-lysine coated dishes (BD Biosciences). After culture for 24 to 48 hours, primary cells were treated with cytosine arabinoside for 24 hours, then changed into fresh NBM media, and cultured in a 37°C cell culture incubator until used for analysis.

### Biotinylation assay

Biotinylation of cell surface proteins was performed as described [26]. Immortalized striatal (STHdhQ7/Q7 and STHdhQ111/Q111) cells were obtained from Coriell and cultured as described [27]. For studying rab11-dependent trafficking of Glut3, STHdhQ7/Q7 were infected with lentivirus expressing permanently active or inactive rab11 for two days and then processed for biotinylation. After biotinylation, cells were washed 3 times in cold 0.1 M Tris/HCl (pH8.0) and scraped into lysis buffer (50 mM Tris/HCl, pH7.4, 150 mM NaCl, 1 mM EDTA, 1% TX-100, and protease inhibitors). Post-nuclear supernatants were incubated with Streptavidin agarose (Pierce). Cell surface proteins (biotinylated) bound on Streptavidin agarose were washed and eluted into SDS-PAGE sample buffer for Western blot analysis. Unbiotinylated (intracellular) proteins in the supernatants were precipitated and resuspended in SDS-PAGE sample buffer for Western blot analysis.

### Immunofluorescence microscopy

Cells on glass coverslips were fixed in 4% paraformaldehyde. After incubation in 50 mM NH_4_Cl containing 0.1% Triton X-100, cells on coverslips were washed, blocked in PBS containing 1% BSA and 0.05% Tween 20, and incubated with antibodies against Glut3 (1: 100, Abcam) and rab11 (1: 100, BD Biosciences) followed by incubation with secondary antibodies conjugated with BODIPY FL (Invitrogen), Cy-3 (Jackson Laboratories) and Hoechst 33258 (Invitrogen). Immunofluorescence microscopy was conducted using a 60 × Oil Nikon Plan Apo objective mounted on an inverted Nikon Eclipse TE300 fluorescent microscope. Images of each channel were collected separately using the Bio-Rad 2100 LaserSharp confocal system and merged using PhotoShop.

Digital confocal images of dual labeled neurons were evaluated for the extent of co-localization of rab11 and Glut3 using NIH ImageJ. Individual neurites from different neurons with clear puncate labeling of the processes were selected for analysis. The number of Glut3 labeled puncta, rab11 labeled puncta and co-labeled puncta were quantified in 20 neurite segments from 20 different neurons. The mean percent ± SD of co-labeled puncta were expressed relative to the total Glut3 labeled puncta and to the total rab11 labeled puncta, respectively.

### Subcellular fractionation

Fresh brains of WT mice (3–6 months old) were minced into pieces in 50 mM Tris/HCl, pH7.4, 150 mM NaCl, 1 mM EDTA containing 10% (w/v) sucrose and protease inhibitors and homogenized by passing brain pieces through a dounce homogenizer for 12 to 15 strokes on ice. Post-nuclear supernatants were supplemented with 60% (w/v) sucrose to a final concentration of 40% (w/v), placed in a SW41 ultracentrifuge tube (Beckman-Coulter), and overlaid with a 10 - 40% (w/v) continuous sucrose gradient. After centrifugation of the gradients at 4°C 100,000 × g for 24 hours, 12 fractions (1 ml each) were collected from the top of the gradient. Equal volumes of each fraction were used for Western blot analysis.

To isolate rab11 positive organelles, brain fractions enriched with rab11 (fraction-5) were diluted in detergent-free immunoprecipitation buffer (final concentration: 25 mM Tris/HCl, pH7.4, 133 mM KCl, 1 mM EDTA, pH7.1) and incubated with antibodies specific for rab11 or FLAG pre-immobilized on protein-A resins. After washes in the detergent-free immunoprecipitation buffer, proteins bound onto protein-A resins were eluted in SDS-PAGE sample buffer and analyzed by SDS-PAGE and Western blot using antibodies against Glut3.

### SDS-PAGE and Western blot

SDS-PAGE and Western blot analysis were performed as described previously [28]. We noticed that Glut3 signals occurred as high molecular weight smears when samples were boiled, and therefore we incubated samples at room temperature for 30 minutes. Protein blotting was performed using the iBlot system (Invitrogen). The blots were blocked in PBS containing 5% nonfat milk and 0.05% Tween-20 and incubated with antibodies against Glut3 (1:500; Abcam) and/or actin (1:500; Sigma-Aldrich) followed by detection with peroxidase-conjugated secondary antibodies (1:5,000; Jackson ImmunoResearch Laboratories). Blots were developed using enhanced ECL (Pierce). Films were scanned. Densitometry was performed on the digitized images using NIH ImageJ.

### Immunohistochemistry detection of cell surface proteins in brain slices and quantitative analysis

A series of coronal sections of 50 μm were cut through the striatum with a vibratome and processed for immunohistochemistry using the immunoperoxidase method. Detergents were absent in all solutions used for labeling and washes to prevent the entrance of antibodies into cells. Brain sections were blocked with 5% donkey serum, 1% bovine serum albumin, and 0.03% hydrogen peroxide in PBS and incubated with goat anti-Glut3 antibodies (I-14, Santa Cruz Biotechnology) or rabbit anti-GABAα1 antibodies (Alomone Labs) at 4°C for two days followed by incubation with biotinylated anti-goat or anti-rabbit IgG(H + L) at 4°C overnight. Brain sections were incubated with ABC reagents (Vector Laboratories, Inc.) at room temperature for 90 minutes followed by DAB staining for 2 minutes. After washes in water, brain sections were mounted on glass slides in 0.5% gelatin and 30% ethanol, and air-dried overnight. After dehydration, brain sections on glass slides were examined and images were collected using a SPOT camera.

Digital images were analyzed using NIH ImageJ by an examiner who was blinded to the conditions. All images were analyzed in the same manner. Glut3 and GABAα1 labeling on cell surfaces was quantified as signal intensity measured using NIH ImageJ. The background was subtracted using a rolling ball radius of 50.0 pixels. The threshold was set with a dark background. The background threshold was set the same for each image analyzed. In some cases, the area to be measured was circled to remove excess staining at the edge of the section. The entire selected area was measured using the “Analyze Particles” Plugin. For neuronal size, the cross-sectional area of each cell was measured using NIH ImageJ. The arbitrary units (A.U.) of signal intensities or cross-sectional areas measured with NIH ImageJ were used for calculating Mean ± SD signal intensity per cell or Mean ± SD cross-sectional area of each cell.

## Results

### Defective trafficking of Glut3 to cell surfaces in primary HD neurons

**Figure 1:**
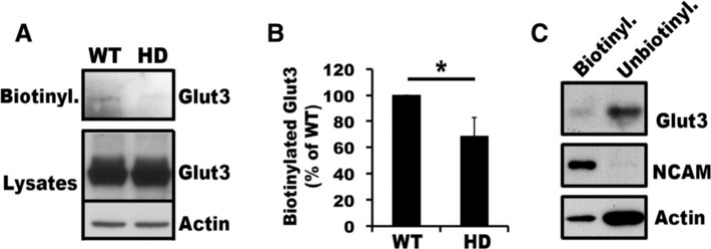
**Impaired trafficking of Glut3 to cell surfaces in primary HD** ^**140Q/140Q**^ **neurons.** Primary neurons at DIV8 were used for studies. **A)** Western blot analysis of biotinylated cell surface proteins and corresponding post-nuclear supernatants of WT and HD cortical neurons. Shown is one blot analysis of three experiments. **B)** Densitometry. Intensities of signals for biotinylated Glut3 (cell surface) and for total Glut3 in post-nuclear supernatants were measured using NIH ImageJ. The ratio of biotinylated Glut3 to total Glut3 was used for calculating the percentage of WT, which was set as 100%. Mean **±** SD percentage of WT was graphed (n = 3 experiments, Student’ ***t***-test: * ***p*** < 0.05). **C)** Western blot shows distribution of Glut3 compared to that of NCAM, a protein known to locate mainly on the neuronal cell surface, and to that of actin, which is predominantly intracellular. Shown is one of 3 sets of analysis of Glut3, NCAM and actin distribution in primary WT cortical neurons. All of the aqueous solution eluted from the Streptavidin beads and 30% of unbiotinylated proteins were used for Western blot analysis.

We previously showed that primary neurons cultured from embryonic cortex of HD^140Q/140Q^ mice took up significantly less glucose than did primary neurons cultured from WT mice [25]. Glut3 is believed to mediate the majority of glucose uptake in neurons. However, the glucose uptake defect in HD^140Q/140Q^ neurons was unlikely to result from aberrant expression of Glut3 protein because the overall levels of Glut3 protein in primary HD^140Q/140Q^ neurons were comparable to those in WT neurons (Figure 1A). To examine if Glut3 trafficking to cell surfaces was altered in neurons of HD^140Q/140Q^ mice, we performed a biotinylation assay to analyze cell surface and intracellular pools of Glut3 in primary neurons. We found that the signal for biotinylated Glut3 was extremely low in both WT and HD^140Q/140Q^ neurons, and was hardly detected in some experiments (Figure 1A and data not shown). This result was consistent with findings by Ferreria and colleagues [29]. Densitometrical analysis of results from three experiments with detectable biotinylated Glut3 signals in both WT and HD^140Q/140Q^ neurons showed that signals for biotinylated Glut3 in HD neurons were reduced by 31.8 ± 14.62% relative to biotinylated Glut3 signals in WT neurons (n = 3 experiments, Mean ± SD, two-tailed Student t-test: *p* < 0.05; Figure 1B). As controls in the biotinylation assay we analyzed neural cell adhesion molecule (NCAM), a cell surface marker of neurons, and actin, a cytoskeletal protein located inside cells, As expected, the majority of NCAM molecules were biotinylated, whereas actin was mainly unbiotinylated (Figure 1C), indicating that cell surface proteins were efficiently biotinylated. Densitometrical analysis revealed that 4.37 ± 2.58% of endogenous Glut3 was located on the cell surface (n = 3 experiments) in WT neurons. Together, these data suggest that Glut3 trafficking to cell surfaces is impaired in HD^140Q/140Q^ primary neurons.

### Glut3 traffics through recycling endosomes

**Figure 2:**
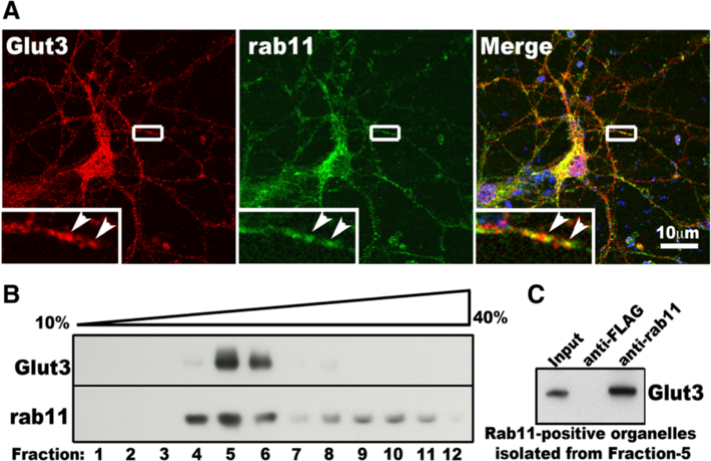
**Glut3 is localized at rab11-positive endosomes. A)**, Co-localization of Glut3 and rab11 in primary cortical neurons. Primary WT neurons on glass coverslips were processed for immunofluorescence labeling of endogenous Glut3 (red) and endogenous rab11 (green) analyzed by confocal microscopy. Yellow areas in the merged photo show signals positive for both Glut3 and rab11. The boxed region containing part of a neurite in each channel is enlarged and shown at the lower left corner of the corresponding channel. Arrowheads in enlarged photos indicate co-localization of Glut3 and rab11. **B)**, Co-distribution of Glut3 with rab11 in subcellular brain fractions. Density gradient ultracentrifugation was conducted as described in Methods. Equal volume of each fraction was used for Western blot analysis. Shown is one blot analysis of three experiments from three mice. **C)**, Glut3 is present in organelles immuno-isolated from brain lysates using anti-rab11 antibodies. Fraction-5 from (**B**) was diluted in detergent-free buffer and incubated with antibodies specific for rab11 or FLAG pre-immobilized on protein-A resins. After washes in detergent-free buffer, proteins on protein-A resins were analyzed by Western blot with antibodies against Glut3. Shown is a Western blot analysis from one of three experiments.

The low abundance of Glut3 on the cell surface is reminiscent of many rab11-dependent trafficking cargo proteins, e.g. transferrin receptor [30]. Therefore, we examined if Glut3 traffics through rab11-positive endosomes in neuronal cells. Primary cortical neurons were double labeled with antibodies for Glut3 and rab11 and confocal imaging analysis was performed as described in methods. Immunoreactivity of endogenous Glut3 and rab11 was seen in punctate structures in neurites and somata (Figure 2A). Some structures were immunoreative for both Glut3 and rab11 (Figure 2A). Quantitative analysis revealed that 35.3 ± 10.7% of Glut3 labeled puncta in the neurites also contained rab11, and 44.4 ± 9.5% of rab11 positive puncta were also positive for Glut3 (Mean ± SD, n = 20 neurite segments from 20 different neurons).

We then performed subcellular fractionation of brain lysates from 3–6 months old WT mice. Western blot analysis showed that Glut3 signal was present in several fractions and peaked in fraction-5, in which rab11 was also enriched (Figure 2B). Immuno-isolation of organelles from gradient fractions was used previously for validating the co-localization of two proteins in the same organelle [31]. Therefore we isolated rab11-positive endosomes from fraction-5 using anti-rab11 antibody. Glut3 was detected in immuno-enriched organelles using anti-rab11 antibody but not in immuno-enriched organelles isolated using anti-FLAG antibody (control antibody) (Figure 2C). These data indicate that Glut3 travels through rab11-positive endosomes.

### Rab11 modulates Glut3 trafficking to the cell surface

**Figure 3:**
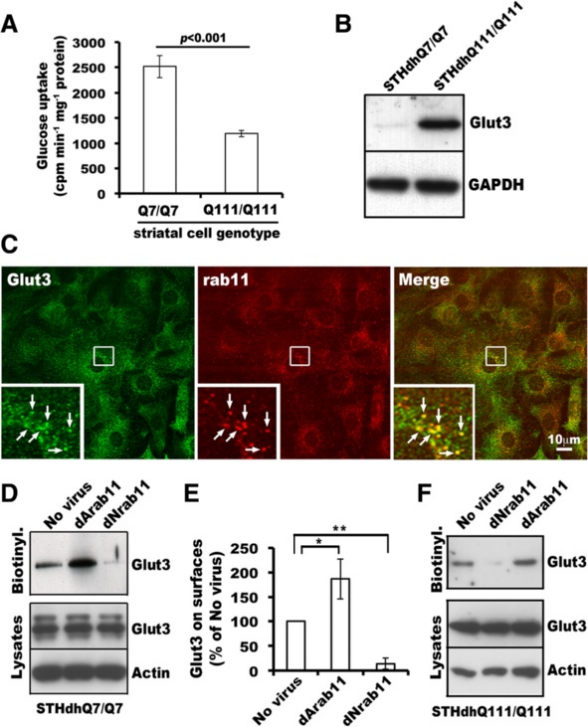
**Rab11 regulates Glut3 trafficking. A)** Defective glucose uptake in immortalized STHdhQ111/Q111 striatal cells. [^3^H]deoxyglucose uptake was performed in PBS at room temperature for 20 minutes. After uptake, cells were washed three times in cold PBS and cell lysates were prepared. The amount of [^3^H]deoxyglucose in cell lysates was measured by liquid scintillation. Mean ± SD cpm per minute per milligram protein was calculated and graphed (N = 3, Two tailed Student *t*-test). **B)** Western blot analysis of post-nuclear supernatants showed up-regulated Glut3 protein expression in STHdhQ111/Q111 cells relative to STHdhQ7/Q7 cells. **C)** STHdhQ7/Q7 cells were cultured on glass coverslips, fixed and processed for immunofluorescence labeling of endogenous Glut3 (green), endogenous rab11 (red), and nuclei (blue). The boxed region is enlarged and shown at the lower left corner of the corresponding channel. Arrows in enlarged photos indicate structures containing both Glut3 and rab11. **D)** Western blot analysis of Glut3 levels upon expression of rab11 mutants. STHdhQ7/Q7 cells in 60-mm dishes were infected with lentivirus expressing dNrab11, or virus expressing dArab11, or uninfected (No virus) for two days. Biotinylated proteins and corresponding total cell lysates were analyzed by Western blot. The results from one of three experiments are shown. **E)** Densitometry of biotinylated Glut3 and corresponding background signals in each condition using NIH ImageJ. The ratio of biotinylated Glut3 signal from cells infected with virus to biotinylated Glut3 signal from cells with no virus infection was graphed (n = 3 experiments, Mean ± SD, Student *t*-test: *p < 0.05; **p < 0.01). **F)** Effects of dArab11 or dNrab11 on cell surface expression of Glut3 in STHdhQ111/Q111 cells. Experimental procedures and data analysis were performed as in **D)**. Shown are blot analysis from one of two experiments.

Having shown the presence of Glut3 at rab11 positive endosomes, we next determined if rab11 regulates Glut3 trafficking. In consideration of technical difficulties to enrich biotinylated cell surface Glut3 from primary neurons, we utilized immortalized striatal cells for investigating rab11 involvement in Glut3 trafficking. Immortalized STHdhQ7/Q7 and STHdhQ111/Q111 striatal cells, which are established from WT and Q111 knock-in HD mouse embryos, respectively [27], are easy to culture and can be efficiently transfected with exogenous genes. Like primary HD^140Q/140Q^ neurons [25], STHdhQ111/Q111 cells were impaired in taking up glucose although total levels of Glut3 protein in STHdhQ111/Q111 cells were about 4.5-fold higher than those in WT STHdhQ7/Q7 cells (Figure 3A and B). In addition, immortalized striatal cells expressed endogenous Glut3, which was co-localized with rab11 and localized mainly inside of cells (Figure 3C). Hence, these cells were excellent tools for studying the role of rab11 in Glut3 trafficking. Dominant negative (dNrab11, rab11S25N) and dominant active (dArab11, rab11Q70L) forms of rab11 are widely used tools for manipulating the function of rab11. We thus examined effects of expressing these rab11 mutants on cell surface expression of Glut3 in STHdhQ7/Q7 cells. Viral expression of either dNrab11 or dArab11 did not affect overall levels of Glut3 protein in STHdhQ7/Q7 cells (Figure 3D). However, levels of biotinylated cell surface Glut3 were significantly increased in cells expressing dArab11 and decreased in cells expressing dNrab11 (Figure 3D and E). Similar effects of expressing rab11 mutants on Glut3 trafficking were also observed in STHdhQ111/Q111 cells (Figure 3F). Taken together, these data indicate that Glut3 trafficking to the cell surface is regulated by rab11.

### Defective Glut3 trafficking in HD brain

**Figure 4:**
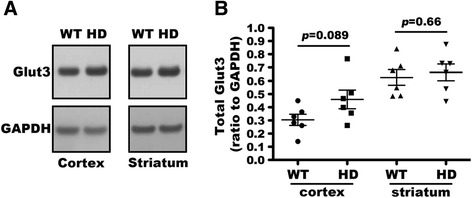
**Normal levels of Glut3 protein in HD** ^**140Q/140Q**^ **mouse brain. A)** Western blot analysis of cortical and striatal lysates of 9–10 month old WT and HD mice. Shown is a blot analysis from one of 6 WT and one of 6 HD mice. **B)** Intensities of signals for Glut3 and for GAPDH in each condition were measured using NIH ImageJ and the ratio of Glut3 to GAPDH signals was calculated (n = 6 WT and 6 HD mice, Mean ± SD, two-tailed Student’s *t*-test).

**Figure 5:**
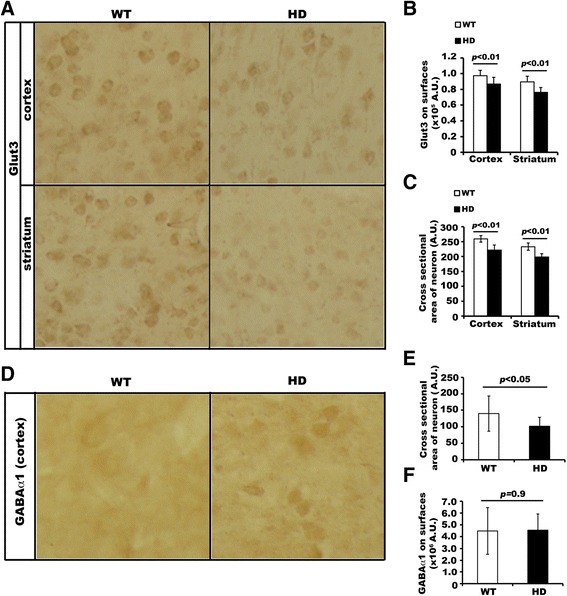
**Defective Glut3 trafficking in the brain of adult HD** ^**140Q/140Q**^ **mice. A)** Antibody binding to detect cell surface Glut3 and GABAα1 in non-permeabilized brain sections of 9–10 month old WT and HD mice. To ascertain that anti-Glut3 and anti-GABAα1 antibodies labeled only cell surface molecules, we omitted detergents in all buffer solutions used for this assay to preserve the intactness of the plasma membrane. Antibodies bound to cell surface Glut3 and GABAα1 were detected using the immunoperoxidase method. Shown in **A** are images of Glut3 labeling obtained with a SPOT camera. Three WT and 3 age- and gender- matched HD mice were used for analysis. **B)** and **C)** Bar graphs show results of densitometry of Glut3 labeled neurons in brain slices for Glut3 surface expression and neuronal cross-sectional area in the cortex and the striatum of adult WT and HD mice. The data were obtained from digital images of 12 microscopic fields of 6 WT and 6 HD brain sections from 2 WT and 2 HD mice, which were treated under exactly the same conditions. The arbitrary units (A.U.) measured with NIH ImageJ were used for calculating Mean ± SD signal intensity per cell. Statistical significance was determined by two-tailed Student’s *t*-test using N = 12 microscopic fields. **D)** Antibody binding to detect cell surface GABAα1 in non-permeabilized brain sections. Brain sections were from the same mice as used for detecting Glut3 expression in **A)**. Shown are images obtained with a SPOT camera. **E)** and **F)** Bar graphs show results for cross sectional areas and signal intensities of GABAα1 positive neurons in the cortex **A**. Digital images were taken from 7 microscopic fields from 4 WT and 4 HD brain sections from 2 WT and 2 HD mice in **(E)** and for GABAα1 signal intensities in **(F)**. Two sections of each animal were used for analysis. For each section, one or two microscopic fields were selected. Error bars in both **E** and **F** represent standard deviations. Two-tailed Student’s *t*-test was performed to determine the statistical significance using N = 7 microscopic fields.

Our above data showed no difference between WT and HD primary neurons in Glut3 protein expression (Figure 1A). We then performed Western blot analysis to determine if overall levels of Glut3 protein expression were altered in the brain of HD^140Q/140Q^ mice. The data in Figure 4 showed that HD^140Q/140Q^ mice at 9–10 months of age expressed levels of Glut3 protein comparable to WT in the cortex and the striatum, indicating that if the cell surface expression of Glut3 was altered in the HD mouse brain it was not a result of reduced overall levels of Glut3 protein expression. To examine if defective Glut3 trafficking occurred in HD brain as it did in HD primary neurons, we performed antibody-binding experiments in non-permeabilized brain slices using antibodies specific for an extracellular epitope of Glut3. We found that Glut3 immuno-reactive signals were significantly reduced by about 11% in cortex and 15% in striatum of HD^140Q/140Q^ brain slices relative to those in WT brain slices (Figure 5A and B). There was also a significant decrease (about 15%) in the cross-sectional area of the HD cortical and striatal neurons compared to those of WT neurons (Figure 5C), indicative of neuronal shrinkage in the HD mouse brain. To determine if the reduced levels of Glut3 on the cell surface of HD neurons was due to neuronal shrinkage or rab11 dysfunction, we examined the surface expression of another protein GABA type A receptor α1 (GABAα1), which has been reported to traffic through a pathway that is not dependent on rab11 [32]. The antibody-binding assay to detect GABAα1 expression on neuronal cell surfaces was conducted in brain slices prepared from the same mice used for detecting Glut3 expression. The cross sectional area of GABAα1 positive cortical neurons was significantly reduced by 27% in HD brain slices (Figure 5D and E). However, the immuno-reactive signals for GABAα1 at the cell surface were comparable in WT and HD brain slices (Figure 5D and F). In the cortex of WT and HD mice, no difference in overall levels of GABAα1 protein was revealed by Western blot assay (N = 6 WT and 6 HD cortex samples compared, data not shown). Together these data indicate that Glut3 trafficking is impaired in the HD mouse brain.

## Discussion

Deficient energy metabolism is a hallmark in the pathogenesis of HD and may arise from multiple factors including reduced glucose utilization in the brain. Studies in immortalized STHdhQ111/Q111 striatal cells suggest that energy deficiency in HD involves an extra-mitochondrial pathway [33]. In agreement with this idea, primary HD^140Q/140Q^ cortical neurons were impaired in taking up glucose, the first step for neurons to utilize glucose. The findings in this study show that rab11, which is known to have diminished activity in HD cells, regulates the cell surface expression of Glut3, the major transporter for glucose uptake in neurons. Relative to WT neurons, HD^140Q/140Q^ neurons expressed normal levels of Glut3 protein, but displayed less Glut3 on the cell surface, suggesting that there is a deficit of Glut3 trafficking to the cell surface in HD neurons. Using an antibody-binding assay to detect protein expression on neuronal cell surfaces in non-permeabilized brain slices of adult mice, we found that the defect of Glut3 trafficking was also present in the brain of adult HD^140Q/140Q^ mice. Our study suggests that a Glut3 trafficking deficit arising from compromised activation of rab11 in HD neurons is a cause of energy metabolism disturbance in HD.

The subcellular localization of Glut3 in neurons was not formally investigated previously. In this study we found that about 4% of Glut3 is located on the cell surface in primary cortical neurons. Ferreira et al. exploited a biotinylation assay similar to ours to investigate Glut3 localization in rat cortical and hippocampal neurons [29]. Although not specifically noted by the authors, a predominantly intracellular localization of Glut3 in neurons is suggested by their data, which revealed extremely low levels of Glut3 on neuronal surfaces at steady state [29]. The mainly intracellular localization of Glut3 is also observed in platelets [34]. However, in other cell types or in conditions of overexpression Glut3 can locate mainly at plasma membranes [12,35]. Why there exists a significant intracellular pool of Glut3 in neurons is not clear. This intracellular pool of Glut3 may allow neurons to quickly respond to conditions demanding high-energy. Transient exposure of cerebellar granule neurons to glutamate vastly increases the expression of Glut3 on the cell surface without altering total levels of Glut3 protein [36]. Stimuli that activate NMDA receptors induce Glut3 translocation from intracellular compartments to cell surfaces [29].

We found that Glut3 was co-localized with rab11 in neurites and somata of primary cortical neurons and was recovered in brain endosomes immuno-isolated using antibodies specific for rab11. Viral expression of permanently active or inactive rab11 respectively altered cell surface expression of Glut3 with no effect on overall expression levels of Glut3 protein. These data suggest that Glut3 in neurons travels through rab11-positive endosomes. As has been proposed for AMPA receptors in hippocampal neurons [37], rab11-positive endosomes may serve as a reservoir for rapid delivery of Glut3 to the neuronal surface in response to stimuli. It is also possible that rab11-positive recycling endosomes act as a station where Glut3 is sorted to its storage compartment in neurons, in a way similar to the sorting of Glut4 to intracellular Glut4 storage compartments in adipocytes and muscle cells [38-40]. Future studies are needed to investigate if such specialized Glut3 storage compartments exist in neurons.

The importance of Glut3 is indicated by studies in mice null for Glut3. Homozygous Glut3 deficient mice die prenatally [41]. While they can survive, heterozygous Glut3-deficient mice show brain developmental characteristics seen in autism spectrum disorders such as abnormal cognitive flexibility with intact motor ability, perturbed social behavior with reduced vocalization and stereotypies, and seizures [42]. We found that Glut3 expression on neuronal cell surfaces was reduced in brain regions that are most affected in HD. It is conceivable that in individuals with HD, a small decline in surface expression of Glut3 occurring over decades could adversely affect glucose uptake and brain function.

## Conclusion

In conclusion, we identify Glut3 as a new cargo protein that is regulated by rab11 during its trafficking to the cell surface and show defective trafficking of Glut3 in HD neurons. This Glut3 trafficking deficit is a result of rab11 dysfunction in HD cells.
